# Enhancing Protein Adsorption Simulations by Using Accelerated Molecular Dynamics

**DOI:** 10.1371/journal.pone.0064883

**Published:** 2013-06-03

**Authors:** Christian Mücksch, Herbert M. Urbassek

**Affiliations:** Physics Department and Research Center OPTIMAS, University of Kaiserslautern, Kaiserslautern, Germany; King’s College, London, United Kingdom

## Abstract

The atomistic modeling of protein adsorption on surfaces is hampered by the different time scales of the simulation (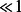 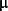
s) and experiment (up to hours), and the accordingly different ‘final’ adsorption conformations. We provide evidence that the method of accelerated molecular dynamics is an efficient tool to obtain equilibrated adsorption states. As a model system we study the adsorption of the protein BMP-2 on graphite in an explicit salt water environment. We demonstrate that due to the considerably improved sampling of conformational space, accelerated molecular dynamics allows to observe the complete unfolding and spreading of the protein on the hydrophobic graphite surface. This result is in agreement with the general finding of protein denaturation upon contact with hydrophobic surfaces.

## Introduction

The interaction of proteins with different biomaterials plays a vital role in describing their biocompatibility. Depending on the surface the overall protein structure may vary from native to unfolded [1]. Especially on hydrophobic surfaces proteins tend to unfold [2]. Experiments on pyrolytic graphite have shown that proteins irrespective of their primary sequence, secondary structure and molecular weight unfold and form nanopatterns [3]. Molecular dynamics (MD) simulations on the other hand provide a direct method for theoretically analyzing the adsorption processes at an atomistic scale.

A major problem that arises when using MD to study protein adsorption is the limited time scale of only a few hundred ns. The experimental time scale when these processes are investigated usually ranges from 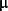
s to hours [4]. It has been shown by Wei et al. that very long simulations are necessary [5] since major adsorption steps like dehydration take a long time and denaturing events even longer. Strategies to overcome this sampling problem are needed. A computationally efficient method using an implicit inviscid water environment has first been established for protein adsorption processes by Raffaini et al. [6], [7]. But Sun et al. [8] argue by comparing implicit and explicit water models that these models show different adsorption behavior with stronger hydrophobic interactions in the case of implicit water.

Based on the work by Voter [9] Hamelberg et al. established an efficient method called accelerated molecular dynamics (in the following abbreviated as: aMD) for biomolecule simulation, which allows to escape the nanosecond time scale limitations [10]. Here, a biasing potential is added to the natural dynamics in order to escape potential energy minima. The method has been applied to many problems like bilayer structural and dynamic properties [11] or the dynamics of bovine pancreatic trypsin inhibitor (BPTI) [12]. Note that it is not straightforward to assign a ‘real time’ to the simulational time due to the bias in the dynamics, especially for rather complicated systems [13].

In the present work, a comparison between classical and accelerated MD is used to show differences in the sampling of conformational changes during adsorption on a hydrophobic graphite surface. To our knowledge, it is the first time that the technique of aMD is used to study protein adsorption. We note that interest in adhesion studies focuses on the final structure and adhesion forces of the adsorbed molecule, since this is where comparison to experiment is possible. The actual adsorption dynamics are not so relevant; this feature makes adsorption a good candidate for aMD.

Our protein of interest is the bone growth factor BMP-2 (bone morphogenetic protein 2) which is used to improve osteointegration by functionalizing implant surfaces [14], [15]. So far, several simulational studies of adsorption of BMP-2 have been performed, but all with classical MD [16]–[19]. To compare the results with previous studies [18], [19] graphite is used as model surface, which has many applications as an implant material [20] in the pyrolytic form.

## Methods

### Simulation Details

**Figure 1:**
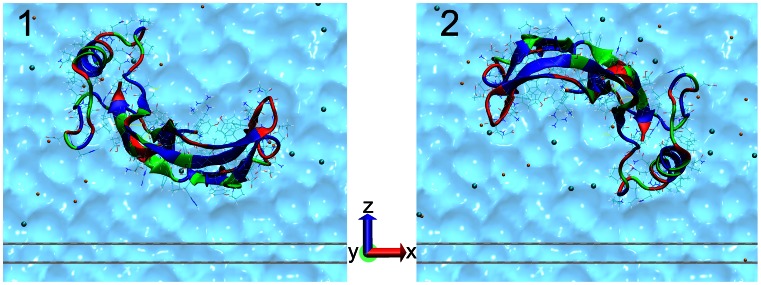
Two different initial orientations of BMP-2 above the graphite surface (indicated by 2 gray lines). Nonpolar residues are shown in blue, neutral residues in green and polar residues in red. The sodium ions are shown in orange and the chloride ions in cyan. The explicit water surrounding is visualized as a volumetric surface in the background.

All the simulations were carried out using NAMD 2.8 [21] with the CHARMM27 force field [22] and the TIP3P [23] water model. The protein structure of BMP-2 was obtained from the protein data base (ID: 3BMP [24]) and placed in two different starting orientations differing by a 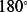 rotation around an axis parallel to the surface so that the lowest protein atom had a distance of 6 Å to the first graphite layer (see Fig. 1).

For all simulations periodic boundary conditions are used with Particle Mesh Ewald (PME) [25] for long range electrostatics. The system is comprised of 43,951 water molecules with a salt concentration of 0.1 M NaCl. Since the protein structure was obtained at a pH of 5.4 and the pI (isoelectric point) of the protein is 4.7 [26] the protein net charge of 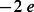 was neutralized for correct usage of PME by having 2 more sodium ions than chloride ions in total. In order to prevent adsorption on the periodic image of the surface the system was chosen sufficiently large with a 25 Å thick layer of water on top of the protein. The cutoff for the van-der-Waals interactions was chosen as 12 Å. To describe the graphite surface [2 layers of (0001)] by Lennard-Jones interactions the needed parameters were taken from Werder et al. [27] to reproduce the experimental contact angle of 86° on graphite [28]. Since these parameters were developed for a different force field and water model we reproduced a 1 ns contact angle simulation with 9000 TIP3P water molecules. This resulted in a contact angle of 85.6° (see Fig. 1) which is in very good agreement with the results by Werder et al. Throughout all simulations the carbon atoms of the surface were kept fixed.

**Figure 2:**
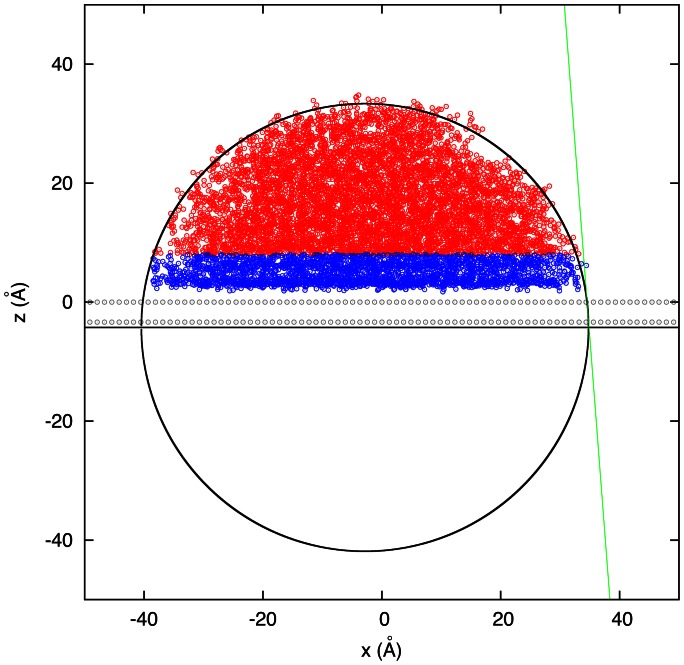
Simulation of water droplet on graphite surface and fit (black circle) through the droplet surface above 8 Å (red) to exclude the near wall region (blue) according to [**27**] **.** The green line represents the tangent to the fitted droplet surface in order to determine the contact angle.

After energy minimization all systems were equilibrated for 100 ps at a temperature of 310 K and constant pressure of 1 atm with the protein backbone atoms restrained in order to prevent adsorption before equilibration. Following the equilibration classical and accelerated MD simulations were carried out for 20 ns with a time step of 2.0 fs enabled by the SHAKE [29] algorithm to ensure rigid hydrogen atoms. Furthermore, orientation 2 was also subjected to a 100 ns simulation run using classical MD.

In order to sufficiently sample the conformational space of adsorption the implementation of accelerated molecular dynamics (aMD) [10] into NAMD [30] was used. In short, a boost potential 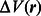 is added to the real potential 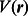 when this is below a chosen value 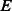 [10]:
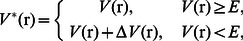
(1)with the boost potential:



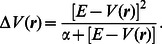
(2)Here a dual acceleration was carried out where the boost potential is applied both to the dihedral potential and to the total potential. This means that besides the protein conformational motion also the diffusivity is increased as the motion of the solvent molecules is also accelerated [31], [32]. The boost parameters were set based upon the average dihedral and potential energy calculated by performing a short classical molecular dynamics (cMD) simulation. They were set according to the recipe given by [31]:
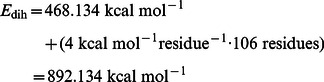
(3)

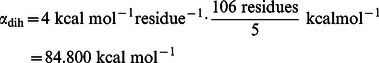
(4)

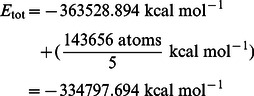
(5)

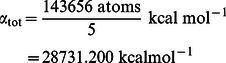
(6)


We also let these simulations run for 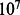 time steps, as done in the cMD simulations. As mentioned in the Introduction, the assignment of a real time corresponding to 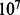 aMD time steps is nontrivial; for brevity we shall in the following also call these aMD simulations to run up to ‘20 ns’.

### Analysis of the Results

The mass weighted radius of gyration is defined as:
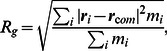
(7)with 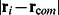 beeing the distance of atom 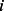 with mass 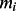 to the center of mass of the protein. In this work we focus on the component parallel to the surface, i. e., 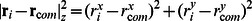, which gives an estimate of the protein spreading on the surface.

Furthermore, principal-component analysis using the cartesian coordinates of the protein atoms was performed with the GROMACS [33] analysis tools in order to describe the conformational sampling of the protein adsorption.

The adsorption snapshots were rendered using VMD [34] and Tachyon [35].

## Results and Discussion

During the first 100 ps both classical and accelerated simulations show a similar trend. The protein approaches the graphite surface driven by attractive van-der-Waals interactions. But very soon classical and accelerated simulations show quite a different adsorption behavior. In the case of aMD, conformational transitions start to occur instantly whereas cMD simulations show very little structural rearrangements after the protein got in contact with the surface. During the accelerated simulations not only the protein motions but also the solvent motions were affected. Since dehydration of the surface is an essential but time consuming step in the adsorption process [5], aMD is able to accelerate the simulated time scale by a considerable factor.

**Figure 3:**
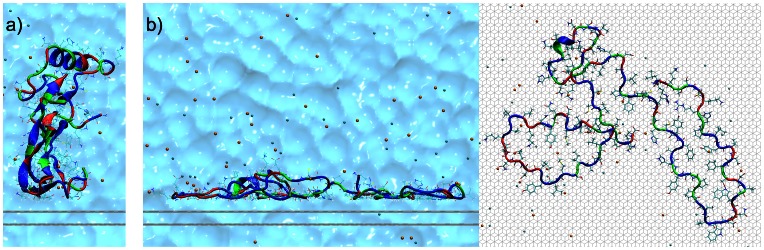
Snapshot of the BMP-2 molecule (orientation 1) after 20 ns. a) cMD; b) aMD with sideview (left) and topview (right).

The final adsorption snapshots after 20 ns, which compare cMD and aMD simulations (Fig. 3), clearly show that accelerated MD has a great impact on the overall structure. A distinctive feature which can be observed is the parallel orientation to the graphite surface of most of the aromatic amino acids (His, Phe, Tyr, Tryp). This finding is in agreement with theoretical work by other authors who investigated amino acid surface interactions [36], [37].

**Figure 4:**
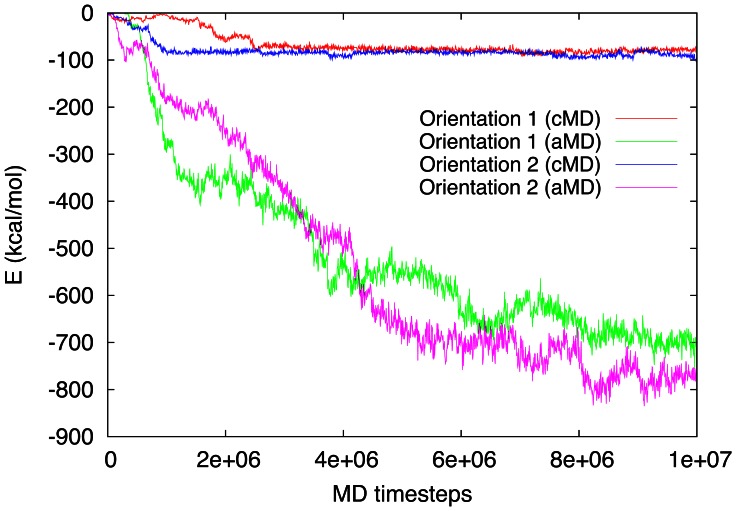
Attractive van-der-Waals energies between BMP-2 and the graphite surface.

Fig. 4 sheds additional light on the improved performance of aMD by displaying the temporal evolution of the van-der-Waals interaction energies between the protein and the graphite surface. Since the surface is neither charged nor polarized in this model we can conclude that van-der-Waals interactions are governing the adsorption process. Note first that indeed the cMD simulation only binds the protein in a shallow metastable precursor state, from which it cannot escape in the 20 ns run; aMD, in contrast, allows for a steady decrease of the binding energy, as the protein continuously changes conformation and acquires deeper bound states. This figure also demonstrates that the number of time steps simulated in this study, 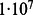 appears sufficient to estimate the final adsorption energy to be around 800 kcal/mol. This is a factor of 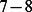 larger than the cMD simulations predict. Note finally that orientation 1 achieves a better binding (larger adsorption energy) than orientation 1, see our discussion of Fig. 4 below.

**Figure 5:**
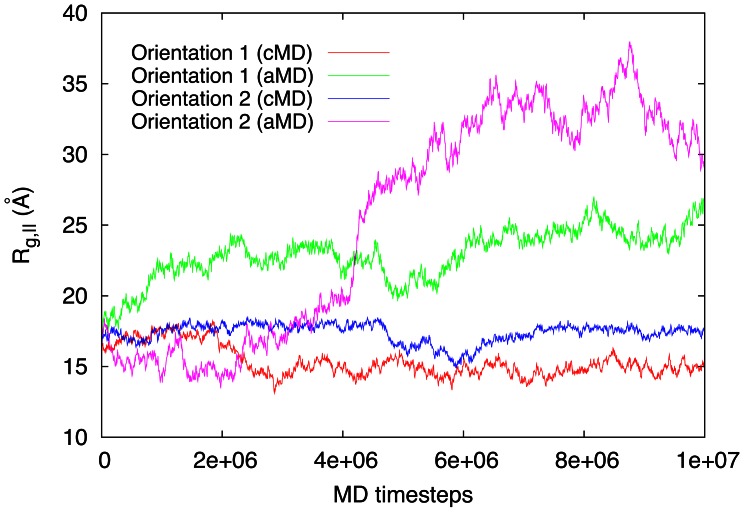
Evolution of the radius of gyration of BMP-2 (component parallel to the graphite surface).

The protein, in the case of aMD, has spread completely on the graphite surface and forms a flat peptide monolayer. This can be confirmed by the temporal evolution of the radius of gyration (Fig. 5). Here, orientation 2 has spread more, both for classical and accelerated MD. The reason hereto is that the molecule has more flexibility in this orientation for spreading out on the surface due to weaker van-der-Waals interactions formed in the early adsorption stage until 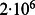 time steps (see Fig. 4); this feature has already been discussed in our previous work [19].

Interestingly, both orientations in the case of aMD show the same secondary structure content after adsorption as can be seen in Table 1. It seems that after 20 ns of accelerated MD simulation using the above mentioned boost parameters the final unfolded adsorption state has been found.

**Table 1:** Secondary structure of BMP-2 before adsorption and at the end of the simulation calculated with dssp [43].

	Initial minimized structure	after 20 ns				after 100 ns
		*Orientation 1*	*Orientation 1*	*Orientation 2*	*Orientation 2*	*Orientation 2*
		*(cMD)*	*(aMD)*	*(cMD)*	*(aMD)*	*(cMD)*
α-helix	15.09%	13.21%	0%	8.49%	0%	11.32%
3_10_-helix	2.83%	0%	5.66%	0%	5.66%	0%
β-sheet	41.51%	34.91%	0%	39.62%	0%	33.02%

When comparing aMD and cMD one can clearly recognize that the helical and 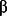-sheet content is only slightly reduced in classical simulations. Even during the 100 ns simulation there has not been a great reduction in the 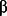-sheet content while one can recognize fluctuations in the helical content comparing with the 20 ns cMD simulation of orientation 2. On the other hand, accelerated simulations show almost no remaining secondary structure except one small 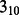-helix. This finding of denaturation is in agreement with other adsorption studies on hydrophobic graphite surfaces [6], [7]. As can be seen in Fig. 3 b topologically distant protein strands show a roughly parallel arrangement which is believed to result from graphite's hydrophobicity, crystallinity, and smoothness [38] and the optimized intramolecular interactions [7]. This phenomenon of unfolded proteins with parallel strands was observed experimentally [3]; this agreement demonstrates that aMD provides reasonable adsorption structures.

**Figure 6:**
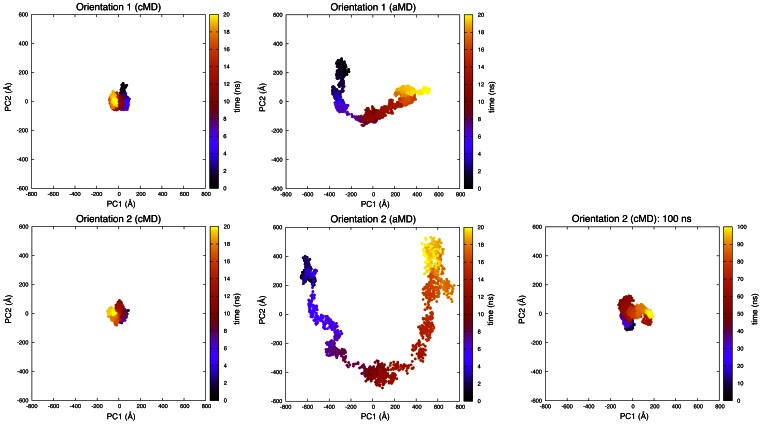
Conformational space obtained by projection of the trajectory onto the first two eigenvectors of the protein atom covariance matrix. Each point represents a conformational state at a simulation time shown by the color code.

Additionally, we performed cartesian principal component analysis to identify the conformational space during protein adsorption (Fig. 6). In this case, the trajectory was projected onto the first two eigenvectors of the protein atom covariance matrix which account for the largest internal motions of the protein. From these results we can clearly conclude that aMD provides considerably improved sampling of the conformational space. This result is in agreement with the findings of investigations using aMD in other applications [10], [12], [30]. Especially in the case of orientation 2 very large conformational motions could be identified. Furthermore, for aMD a clear unfolding pathway can be seen for both orientations where many conformational states were visited. Extending the classical adsorption simulation to 100 ns resulted only in a slight extension of the visited conformational space. Other results employing long classical simulations (300 ns) suggest that even after long simulation times, which seemingly equilibrated the system, sudden structural changes may occur [5].

## Conclusions

Comparing our results obtained by classical and accelerated molecular dynamics simulations we conclude that sufficient sampling of the conformational space is key to understanding protein adsorption. Since adsorption phenomena take place in time scales extending from 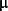
s to hours [4], the use of classical MD simulations of a few nanoseconds up to 100 ns in an explicit water surrounding is not adequate for studying and describing the entire adsorption process. Our approach uses an explicit water environment with standard temperature and pressure control. By modifying the potential energy landscape through accelerated MD, conformational changes can occur in the time range accessible to simulation. Especially during adsorption the protein can get stuck in metastable states that hinder further spreading on hydrophobic surfaces.

The advantages of this method are – among many others (see [10]) – (i) the potential for application in many different surfaces ranging from hydrophobic to hydrophilic, (ii) the realistic system setup (e. g. inclusion of ions, atmospheric pressure) with no need for very long energy minimizations, (iii) a faster relaxation of water molecules [32], and finally (iv) only minor additional computational effort (see Figure 1 in [30]). Beside many available advanced sampling methods, recently reviewed by Zuckerman [39], we would like to point out an approach by Wang et al. [40] which uses biased replica-exchange molecular dynamics (REMD) to study adsorption processes. Comparing biased-REMD to accelerated MD we emphasize that aMD, besides being easy to handle, is computationally more efficient since no different replicas have to be simulated; the latter feature limits biased-REMD to smaller systems. On the other hand, REMD will give exact dynamics with a known reweighting factor.

The easiness of use and computational efficiency make aMD an ideal candidate for studying adsorption processes in large systems. Other interesting sampling techniques such as conformational flooding [41], [42] might be used as well to address adsorption studies but appear not so straightforward as aMD. Conformational flooding for instance needs calculation of a ‘flooding matrix’ and possibly multiple flooding potentials. Finally, coarse-grained models that might provide an interesting alternative for calculating large systems over long time scales always bear the risk of producing artifacts.

Our results suggest that the monomeric form of BMP-2 does indeed unfold on a hydrophobic graphite surface as suggested by previous results [19]. Despite the different protein structure (monomer vs. bioactive dimer [24]) this is in clear contrast to recent results by Utesch et al. [18]. It appears logical that a classical 10 ns MD simulation, such as that performed in [18], cannot provide insight into structural rearrangements during adsorption since the conformational space is sampled only insufficiently. We note that even in cMD simulations extending over several hundred ns, it has been found that the final configuration is governed by sudden conformation jumps which may occur late in the simulation [5].

Even though the dynamics of the adsorption process have been altered, the final adsorption state is most relevant in adsorption studies, since it can be compared with experimental results. From what we know from experimental results [3], e.g. an unfolded protein structure or the formation of parallel protein strands, the adsorption state obtained by aMD provides a considerably more realistic prediction than the ‘unfinished’ adsorption state after 20 ns of classical simulation. The next step in characterizing protein adsorption processes will be the utilization of aMD for studying a variety of different protein-surface interactions including initial orientation and topology dependences. Comparison to experiment is for instance possible by an analysis of the secondary structure content of the adsorbed protein or by determining the forces necessary for protein desorption.
